# The effectiveness of acupuncture in cancer pain treatment

**DOI:** 10.3389/fonc.2024.1450105

**Published:** 2024-11-21

**Authors:** Kamila Krukowska, Sylwia Krzyśkowska, Eliza Kuchta, Anna Rudzińska, Katarzyna Szklener, Sławomir Mańdziuk

**Affiliations:** Department of Clinical Oncology and Chemotherapy, Medical University of Lublin, Lublin, Poland

**Keywords:** acupuncture, pain management, cancer pain, quality of life, complementary medicine

## Abstract

**Introduction:**

In recent years, pain has been recognized as a primary factor significantly diminishing the quality of life in cancer patients. Recent data have prompted the establishment and increased application of non-pharmacological interventions in pain management, such as acupuncture.

**Objectives:**

This review assesses literature from 2018 to 2023 on the impact of acupuncture on pain management and quality of life in cancer patients, with a particular focus on reducing pain intensity. The effectiveness of acupuncture therapy was compared with the traditional treatment of pain symptoms, with a focus on the patients’ quality of life.

**Conclusions:**

Although no conclusive scientific evidence confirms the effectiveness of acupuncture in treating cancer pain symptoms, numerous studies have demonstrated its ability to reduce pain, better control pain, decrease analgesic intake, and significantly improve patients’ quality of life. Further research is needed to unequivocally confirm the clinical benefits of acupuncture.

## 1 Introduction

Pain is one of the most distressing symptoms accompanying cancer. It hinders patients’ daily activities and significantly impairs their quality of life (1, 2). According to recommendations, the treatment of cancer-related pain should be based on a combination of various methods, including the use of opioid and non-opioid medications, manual therapies, and psychotherapies (1, 2). Although the analgesic ladder recommended by the World Health Organization provides guidelines for alleviating cancer-related pain, the dependence on pain-relieving drugs and the negative side effects of pharmacotherapy pose significant challenges for both patients and physicians (1, 2). In line with increasing clinical evidence of alternative therapies (3), leading medical organizations recommend the use of non-pharmacological interventions, including acupuncture (4).

Acupuncture is an increasingly popular method of pain management that can be used as an adjunctive therapy or an integral part of a treatment plan (4). This form of therapy is well-tolerated and carries a low risk of adverse effects. Both traditional acupuncture and unconventional methods, such as electroacupuncture, ear acupuncture, thermal acupuncture, or laser acupuncture, often result in a reduction of perceived pain (4). There are several factors that influence the effectiveness of this therapy, including needling technique, the number of needles used, the duration of the procedure, precise acupuncture point placement, the number of sessions conducted, and subjective factors such as the patient’s psychological approach (4).

## 2 Mechanism of acupuncture

Acupuncture involves inserting needles at specific points on the body to alleviate one or more symptoms (5). These points are individually selected for each patient based on the symptoms that need relief (5). Mechanisms of action include an increase of the production of endorphins and adenosine and regulation of the matrix responsible for the pain perception (6). Acupuncture has been found to cause an immunomodulatory effect in cancer patients by inducing significant changes in their blood, including an increase in interleukin-2, natural killer cells, and CD3+ and CD4+ T lymphocytes (7). Increasing their levels in acupuncture therapy is crucial for pain relief. IL-2 is essential for the growth, proliferation, and differentiation of T cells, which contributes to enhancing the immune response to tumors. CD3+ and CD4+ T cells play a significant role in activating and regulating the immune response, which can lead to more effective pain management and reduced inflammation. Additionally, NK cells have the ability to eliminate cancer cells, which can improve patients’ quality of life by alleviating pain-related symptoms. Therefore, the increase in these cells and cytokines is associated with an improvement in the overall health and well-being of patients (7).

Modern science provides a growing body of evidence regarding the biological mechanisms of acupuncture. These findings suggest acupuncture stimulates reflexes that activate peripheral nerves, transmitting sensory information to the brain via the spinal cord (8). Subsequently, this activation influences the autonomic nervous system and affects various physiological processes within the body (8). Acupuncture reduces the negative impact from chemotherapy or radiotherapy and enhances the quality of life, alleviates symptoms associated with cancer-related fatigue, and delays cancer progression (9–12). According to numerous scientific studies, acupuncture influences symptoms such as pain, nausea, depression, fatigue, anxiety, decreased well-being, shortness of breath, and drowsiness (13–15). These symptoms were primarily considered in the assessment of acupuncture efficacy (13).

The molecular mechanism of acupuncture and electroacupuncture (EA) is not fully understood, but there is little evidence of how these therapies affect the body (14). Studies have shown that acupuncture may influence the actions of central chemical mediators, such as neurotransmitters (16). An example is naloxone, a specific antagonist of opioid receptors, which partially reverses the analgesic effects of acupuncture (16).

Furthermore, research indicates that acupuncture and EA may influence the action of substance P (SP) (17), which is released during inflammatory states in the nervous system and increases the activity of sensory fibers leading to acupuncture points (18). These therapies can reduce the expression of substance P, which plays a significant role in pain signal transmission (14). Electroacupuncture may affect the serotonin system by increasing the expression of 5-HT2A/C receptors (14, 17). Additionally, EA can reduce mitochondrial oxidative stress and increase ATP production, which helps alleviate chronic fatigue (16). Acupuncture and EA also affect the sympathetic nervous system and the hypothalamic-pituitary-adrenal axis, which can have a beneficial impact on the body (16). Studies suggest that these therapies may influence the secretion of various hormones and peptides, such as ghrelin, cholecystokinin (CCK), and 5-hydroxytryptamine (5-HT) (16).

This way, they bridge traditional techniques with a modern understanding of mechanisms, offering hope for improving the health and well-being of patients (14).

This work focuses on the specific aspects of acupuncture’s application in alleviating cancer-related pain in patients with various types of cancer. The main focus on pain associated with gastric, lung, breast, colorectal and prostate cancers, which are among the most common cancer forms worldwide (19). We analyzed scientific research on the effectiveness of acupuncture in relieving cancer-related pain and presented the findings on acupuncture impact on patients’ quality of life.

## 3 Acupuncture in cancer

### 3.1 Gastric cancer

Stomach cancer (GC) is a significant global health issue. Its incidence and mortality rates vary significantly among countries (20).

**Figure 1:**
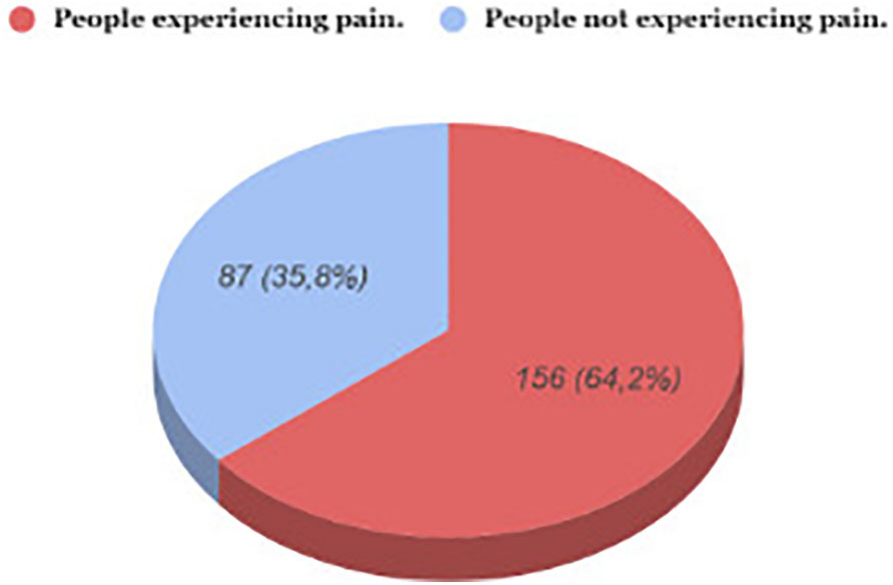
The experience of pain by the examined patients.

GC significantly impacts the quality of life of patients, and the issue of pain, arising from both the disease itself and the employed therapeutic procedures, poses a substantial clinical challenge. A 2021 study, conducted with the participation of 243 patients diagnosed with stomach cancer, revealed the pain as the most commonly reported ailment, experienced by 64.2% patients. (**Figure 1**) (21).

A clinical trial conducted in China from January 2019 to February 2020 in seven different Chinese hospitals researched the effectiveness of electroacupuncture (EA). The analysis involved a sample of 58 patients undergoing adjuvant chemotherapy using the CapeOx (Oxaliplatin + Capecitabine) or SOX (Oxaliplatin + S-1) regimen. Patients were randomly assigned to either a control group or a group that received EA two or one time(s) per week for six weeks. To assess The Health-Related Quality of Life (HRQOL), the Chinese version of the Functional Assessment of Cancer Therapy-Gastric (FACT-Ga) questionnaire was utilized. In this pilot study, the GaCS subscale was particularly statistically significant, as it focuses on issues directly related to gastric cancer (22).

The results showed that patients receiving EA had significantly higher HRQOL scores and experienced fewer disease-specific symptoms than patients in the control group. The mean score on the GaCS for patients receiving EA therapy was 51.98, with a standard deviation of 10.91. In contrast, patients in the control group achieved a mean score of 45.37, with a standard deviation of 8.61 (**Table 1**). Statistical analysis revealed a significant difference between these two groups, with a P-value of 0.039. However, no significant differences in outcomes were observed between the group receiving a high dose of EA (twice a week) and the group receiving a low dose of EA (once a week), suggesting that the frequency of acupuncture application lacked a significant impact on the study’s results (22).

**Table 1:** GaCS scores.

GaCS scores	EA group (n=28)	Control group (n=17)
Baseline before chemotherapy ± SD	52.95 ± 10.97	49.98 ± 10.62
Average during chemotherapy ± SD	51.98 ± 10.91	45.37 ± 8.61

The table presents the data as the mean ± standard deviation (SD). Higher Gastric Cancer Scores (GaCS) indicate a more favorable outcome for patients in the electroacupuncture (EA) and control groups (22).

**Figure 2:**
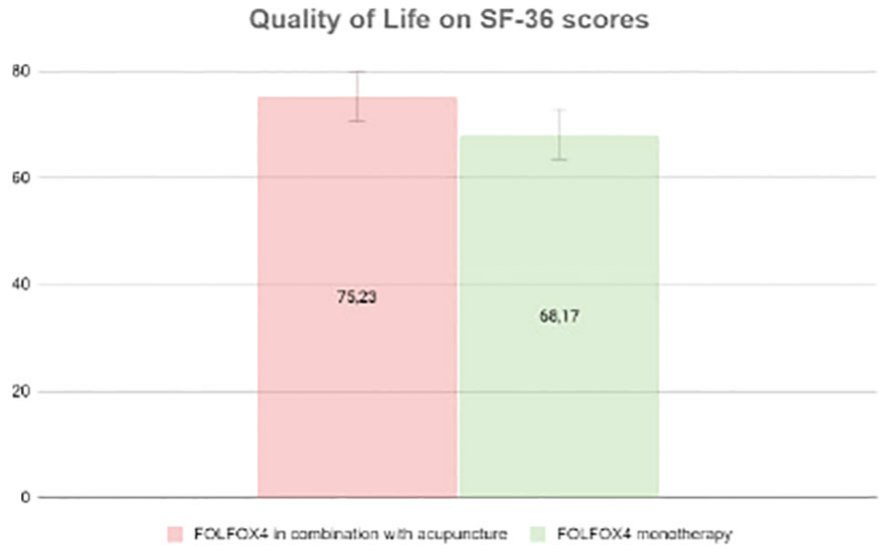
SF-36 Score.

In a study recruiting 120 advanced gastric cancer patients the effectiveness of acupuncture as an additional therapy for patients receiving the FOLFOX4 chemotherapy regimen (comprising oxaliplatin and 5-fluorouracil) was researched. The study lasted in the period from May 2019 to April 2021 and outcome measures included levels of tumor markers, adverse events during therapy, and the quality of life of the patients. "A 36-point abbreviated version of the Medical Outcomes Study 36-Item Short Form Health Survey (SF-36) was used to assess the patients’ quality of life, with physical pain being one of the aspects evaluated (**Figure 2**) (23).

The study also confirmed that acupuncture can alleviate many side effects associated with chemotherapy. Additionally, the research showed that acupuncture may influence the regulation of tumor markers in patients, indicating its effectiveness in combating the disease (23).

The aim of another 2017 study was to assess the effectiveness of acupuncture in alleviating gastrointestinal symptoms associated with chemotherapy. The study involved 56 patients receiving chemotherapy with oxaliplatin and paclitaxel. The diagnostic parameters evaluated included the level of abdominal pain, the patient’s quality of life, the safety of acupuncture, and the average duration and cost of hospitalization. The study also took into account the duration of nausea, the frequency of vomiting and diarrhea. In both experimental and control groups, patients reported experiencing pain lasting an average of 7 ± 2 and 16 ± 5 minutes per day, respectively, indicating the efficacy of pain relief associated with the oncological condition. Furthermore, acupuncture helped reduce symptoms of nausea, vomiting, and diarrhea (24).

Postoperative ileus (POI) is a common complication of abdominal surgery, including gastric operations. POI is characterized by the obstruction or impairment of the passage of the digestive content through the intestines and presents with symptoms such as severe abdominal pain (25–29).

In a 2018 study, participants were divided into four groups receiving different forms of therapy at the ST36 acupuncture point, located on the lateral side of the leg. The first group received acupuncture, the second group received an ST36 acupoint injection with neostigmine, the third group received intramuscular neostigmine injection, and the fourth group underwent standard therapy. Patients were auscultated to monitor bowel sounds to determine the therapy effectiveness. Adverse events, including abdominal pain, were monitored using a questionnaire. The main outcome measure was the effectiveness rate, defined as the first occurrence of relief from bloating or the first bowel movement within an hour after treatment (26).

In the group receiving ST36 acupuncture, the effectiveness was 6.77%, in the group with ST36 acupoint injection, it was 94.03%, and in the intramuscular injection group, it was 64.18%. The group receiving standard therapy achieved an effectiveness rate of 3.78% (**Table 2**). It is worth noting that the ST36 acupuncture group had a lower level of abdominal pain and treatment-related side effects (26).

**Table 2:** Effectiveness rate.

Variable	Total effectiveness (%)
Acupuncture (n = 59)	4 (6.77)
Acupoint injection (n = 67)	63 (94.03)
Intramuscular injection (n = 67)	43 (64.18)
Standard therapy (n = 53)	2 (3.78)

Two other studies focused on the impact of acupuncture on postoperative intestinal obstruction: a study conducted at the Cancer Hospital Chinese Academy of Medical Sciences between May 2018 and August 2019 (Research 1) and a study conducted at the Daegu Catholic University Medical Center from March to December 2015 (Research 2). The results, outcome measure, and the significance of acupuncture of these respective studies are in the table below (**Tables 3**, **4**) (27, 28).

**Table 3:** Study conducted at the Cancer Hospital, CAMS (Research 1).

Characteristic	Research 1		
Number of patients	385		
Acupuncture information	EA Group (Gr. 1) n = 201	EA before and after surgery	
	Control group (Gr. 2) n = 184	Placebo needle and fake electric needle	
Main Outcome Measure	Time (in days) from surgery to the first occurrence of bloating and defecation.		
Results	Defecation (open surgery/laparoscopy) ^a^	Gr. 1	3,1 ± 0,7/2,8 ± 0,7
		Gr. 2	4,2 ± 1,1/5,5 ± 1,2
	Defecation (open surgery/laparoscopy) ^a^	Gr. 1	4,3 ± 0,9/3,9 ± 0,9
		Gr. 2	3,9 ± 0,9/5,1 ± 1,1

a - values provided in days ± SD (standard deviation).

**Table 4:** Study conducted at Daegu Catholic University Medical Center (Research 2).

Characteristic	Research 2		
Number of patients	36		
Acupuncture information	Acupuncture Group (Gr. 1) n=18		Acupuncture once a day for 5 days after surgery + perioperative management
	Control group (Gr. 2) n = 18		perioperative management
Main Outcome Measure	The number of sitz markers remaining in the small intestine, unpassed through the ileocecal valve.		
Results	Day 3 ^b^	Gr. 1	3,22 ± 4,26
		Gr. 2	14,17 ± 4,02
	Day 5 ^b^	Gr. 1	0,00 ± 0,00
		Gr. 2	5,89 ± 3,18

b - Values are presented as the mean number of visible Sitz markers in the intestine ± SD (standard deviation).

In a 2019 study, it was observed that among individuals undergoing open surgery, the time to first flatus and defecation in the EA group was significantly shorter than in the control group. The same result was noted in laparoscopic surgery (27).

In the Daegu Center study, it was observed that on the third day post-operation, the majority of the Sitz markers had passed through the ileocecal valve (IC) in patients who underwent acupuncture. In the control group, only slight displacement of Sitz markers within the small intestine was noted, with none of them crossing the IC valve. By the fifth day, in the acupuncture group, the presence of Sitz markers in the intestines was no longer observed (28).

TEAS (Transcutaneous Electrical Acupoint Stimulation) is a non-invasive method that involves stimulation of acupuncture points by electrodes. The study was conducted at the Affiliated Hospital of Qingdao University from May 1, 2019, to October 31, 2019, and involved 82 patients. The patients were randomly assigned to two groups in a 1:1 ratio (**Table 5**). The experimental group, which underwent TEAS, received auricular electroacupuncture within three days after the surgery (from the first to the third postoperative day). The therapeutic procedure was administered twice a day, at 8:00 AM and 4:00 PM. The whole process took 30 minutes during each session (29).

**Table 5:** Patient characteristics.

Characteristic	Study Group	Control Group
Number of participants	41	40
Women, n (%)	14 (34,1%)	12 (30%)
Men, n (%)	27 (65,9%)	28 (70%)
Mean age (years)	58,71	60,83
Distal resection, n (%)	30 (73,2%)	35 (87,5%)
Total resection, n (%)	11 (26,8%)	5 (12,5%)
Abdominal pain experienced before resection and TEAS therapy, n (%)	26 (63,4%)	26 (65%)

One of the key indicators examined was the assessment of pain intensity over a five-day period following gastrectomy surgery. Additionally, the quantity of pain-relief medications, including opioids, was assessed (29).

The results of the conducted research have shown that the use of TEAS led to a significant alleviation of pain symptoms, reducing them by approximately 20%. Furthermore, a significant reduction in the consumption of non-steroidal anti-inflammatory drugs (NSAIDs) by 17.8%, opioids by 31.1%, and an equivalent dose of morphine by around 3.9 mg was noted (**Table 6**). Consequently, the average consumption of morphine by patients undergoing TEAS therapy reduced by nearly half, which was associated with less postoperative pain intensity (29).

**Table 6:** Main results.

Indicators	Study Group n=41	Control Group n=40
Average pain for 1-5 days after surgery ± SD	2,55 ± 0,21	3,10 ± 0,42
Opioid utilization rate, n (%)	18 (43,9%)	30 (70%)
Equivalent morphine dose (mg), mean ± SD	8,935 ± 3,660	12,83 ± 8,097

Numerical Rating Scale (NRS) was employed for pain assessment, integrated with the Wong-Baker Faces Pain Rating Scale.

### 3.2 Breast cancer

Currently, according to the latest WHO data, breast cancer is the most commonly occurring cancer worldwide. In 2020, there were 2.26 million cases of breast cancer, making it the fifth leading cause of cancer-related deaths globally (30).

Regardless of the stage of breast cancer, besides pharmacological and conventional treatment methods, non-pharmacological therapies such as mind-body practices, acupuncture, massage, music therapy, hypnosis, meditation, or yoga are recommended. These methods are still relatively under researched however, the available evidence suggests their effectiveness, particulary when used with traditional treatment. Alternative forms of therapy, can provide comprehensive care for patients and mitigate the side effects of the conventional treatment, in particular in breast pain management (31).

In 2021, a comprehensive meta-analysis was conducted, classifying patients into five subgroups based on symptoms caused by drug therapy (gastrointestinal disturbances, chemotherapy-induced peripheral neuropathy, joint pain associated with aromatase inhibitors, joint symptoms associated with aromatase inhibitors, and cognitive impairments). A total of sixteen randomized controlled trials with 1189 participants were included in the meta-analysis. The main result and assessments of various subgroups demonstrated a statistically significant enhancement in managing side effects through authentic acupuncture. The patients’ quality of life improved during the treatment (32).

A comprehensive analysis of research conducted until 2018 showed that acupuncture can help with pain management in breast cancer patients. The number of participants in the studies included in the meta-analysis ranged from 32 to 190, all of whom were breast cancer patients. The average age of the participants ranged from 40 to 60 years. Breast cancer-related symptoms included: menopausal symptoms (8 articles), pain (4 articles), nausea and vomiting (2 articles), lymphedema (2 articles), peripheral neuropathy (1 article), cognitive impairments (1 article), and gastrointestinal symptoms (1 article). During the studies, chemotherapy, aromatase inhibitor therapy, hormonal therapy, surgery, and symptomatic treatment were mentioned. In studies focusing on patients experiencing pain caused by anticancer treatment, significant differences were observed after acupuncture treatment. Pain was assessed using the Western Ontario and McMaster Universities Arthritis Index (WOMAC), Brief Pain Inventory-short form (BPI-SF), and Functional Assessment of Cancer Therapy–General (FACT-G) (33).

Recent studies revealed that approximately half of the patients diagnosed with cancer use unconventional therapies and achieve high satisfaction levels. A qualitative study was conducted involving 20 individuals to examine the impact of acupuncture on the adverse effects of chemotherapy in breast cancer patients (34). All patients in the acupuncture group reported an improvement in acute physical and psychological symptoms after acupuncture. A reduction in headache, limb pain, gastrointestinal issues, and polyneuropathy was noted by most patients, except one who did not report a reduction in physical discomfort. All respondents also observed an improvement in their mental state, expressing a greater sense of relaxation, reduced stress, less anxiety, and better sleep after undergoing acupuncture (34).

Similarly, another study conducted by the Mayo Clinic that significantly influenced the evaluation of the effectiveness of acupuncture in alleviating the effects of the disease or the side effects of its treatment was a cross-sectional survey in which 415 individuals participated. Of the 241 women who underwent acupuncture, over 80% reported benefits from acupuncture, while more than half of the respondents reported a reduction in the average severity of symptoms. Only 5% of patients reported negative side effects of the therapy, such as discomfort. According to some participants who underwent acupuncture, their headaches decreased. Specific key findings of the study are presented below (**Table 7**) (35).

**Table 7:** Results of the study conducted by the Mayo Clinic.

Symptoms	The number of individuals % (n)^a^	Improvement %^b^
Joint Pain	87 (36,1)	73
Muscle Pain	83 (34,4)	66
Post-surgical pain	45 (18,7)	68
Headache	35 (14,5)	79
Neuropathy	61 (25,3)	69

a - The percentage of women experiencing a particular symptom. b - The percentage of patients who reported an improvement in symptoms.

### 3.3 Lung cancer

Lung cancer (LC) is a malignant cancer and according to data from 2023, it is the most common cause of death worldwide among all types of cancer (36, 37). One of the most aggravating symptoms of lung cancer is pain, which significantly affects the quality of life of patients. As a result, unconventional treatment options directed towards combating lung cancer and its symptoms are being explored, including acupuncture (37).

Acupuncture may reduce the amount of opioids taken after surgery and improve their analgesic effect (37). A 2021 study was conducted to evaluate the effectiveness and safety of acupuncture combined with analgesics for the treatment of lung cancer-related pain (37). The study included participants who had this type of pain, regardless of gender, race or stage of disease. The results were analysed in terms of analgesia, pain intensity and possible adverse reactions (37). The above study is still in the recruitment phase (37).

A randomized, double-blind study with 80 participants after thoroscopic lung removal aimed to determine the efficacy of transdermal electrical acupuncture stimulation (TEAS) for analgesia and postoperative sedation in patients with lung cancer (16). Control group received acupuncture and the study group was subjected to the TEAS treatment. Postoperative pain was assessed at 6, 24, 48 hours and one month after surgery using a visual analogue scale (VAS) (16).

The patients in the study were between 18 and 64 years old and it was their first lung surgery. In addition, the subjects had never undergone acupuncture and were not burdened with other severe coexisting diseases (16).

In this study, it was observed that the results on the VAS scale were significantly lower in patients who underwent acupuncture compared to the group that received simulated treatment. Specifically, the results after one month showed a significant difference, with VAS scores being much lower in patients receiving acupuncture. There was also a lower consumption of sufentanil in patients who received acupuncture, which was used for analgesia and pain relief. Additionally, patients who underwent acupuncture experienced reduced nausea and vomiting after surgery compared to those who underwent simulation (**Table 8**) (16).

**Table 8:** Characteristics of the test.

Test criteria	Data
Number of patients	80 patients
The characteristics of patients	Patients between the ages of 18 and 64, following the first surgical procedure, without prior contact with acupuncture methods
The main analytical criterium	postoperative pain level (mainly)
The time after which the level of postoperative pain was measured	6h, 24h and 48h and a month after surgery
Scale used to measure postoperative pain	Visual Analog Scale (VAS)

In a 2022 meta-analysis conducted by Dan Wang and contributors, 16 studies involving a total of 1305 patients were evaluated, with 651 in the TEAS group and 654 patients in the control group (38). The primary objective of this meta-analysis was to assess postoperative pain on the VAS scale, and the secondary objectives were the amount of opioids consumed and potential adverse events occurring within 24–72 hours after surgery (38). The use of TEAS was strongly associated with a reduction in VAS scores. The use of painkillers was also decreased in the acupuncture group. In addition, the incidence of nausea, dizziness and vomiting in patients after TEAS was significantly lower (**Table 9**) (38).

**Table 9:** Patient results after TEAS.

Criterium	Scales and values after teas
VAS 24h after surgery	open surgery: SMD = −0,84, 95% CI = −1,07∼−0,6, I2 = 96%; minimally invasive surgery: SMD = −0,88, 95% CI = −1,02∼−0,75, I2 = 96%
Frequency of dizziness after surgery	RR = 0,48, 95% CI 0,34∼0,68, I2 = 0%
Painkillers 72h after surgery	SMD = −2,10, 95% CI = −3,37∼−0,82, I2 = 96%
Frequency of postoperative nausea	RR = 0,66, 95% CI 0,44~1,01, I2 = 69%
Frequency of postoperative vomiting	RR = 0,49, 95% CI = 0,24∼1,00, I2 = 51%

### 3.4 Colorectal cancer

Colorectal cancer poses a significant health challenge and is the second leading cause of cancer-related deaths worldwide (39).

The surgical removal of colorectal cancer poses a risk of postoperative bowel obstruction leading to complications, pain, and an extended hospital stay. In such cases, electroacupuncture is sometimes used as an alternative method for treating gastrointestinal dysfunction, although its effectiveness is still a subject of research (40).

A study conducted at the Second Affiliated Hospital of Xi’an Jiaotong University on patients after the resection of colorectal or gastric cancer demonstrated the beneficial impact of acupuncture on treatment. In this study, 64 patients were divided into two groups, one of which received postoperative acupuncture. The results of the study revealed significant differences in the time to the first postoperative bloating, bowel movement, and oral feeding, as well as in alleviating gastrointestinal symptoms, suggesting the favorable influence of acupuncture on the postoperative recovery of colorectal cancer patients (25).

The latest meta-analysis, encompassing 1878 studies involving 876 patients, focusing on the use of acupuncture in patients after colorectal cancer resection, indicates potential benefits of acupuncture. These benefits include pain and nausea reduction, improved bowel function, and overall physical and emotional well-being. Nevertheless, there are research directions that remain to determine the optimal timing and duration of acupuncture therapy (41).

Another meta-analysis, including 42 relevant articles, also showed that acupuncture therapy is significantly more effective in improving gastrointestinal function compared to standard postoperative care. No serious adverse events related to acupuncture were reported in the studies. Additionally, it emphasized the significant role of acupuncture points on the lower limbs, especially the He-sea point, in the context of treating POI (41).

### 3.5 Prostate cancer

Prostate cancer (PC) is one of the most common malignant cancers in men worldwid (42, 43). Patients suffering from this type of cancer often experience significant pain, due to the prostaglandins secretion caused by the disease, which hypersensitive the nerve endings around the tumor (44). According to American researchers, prolonged use of painkillers can cause the spread of cancer cells and tumor growth (44). As a result, the interest in alternative methods of PC management, including acupuncture, is increasing.

Multiple studies are being conducted on the effect of the acupuncture on prostate cancer, but many of them are still in the recruitment phase. In a study initiated in 2021, the effectiveness of acupuncture was compared to observational or standard care in the treatment of hot flashes induced by antiandrogens in prostate cancer (45). The study included patients aged 18 to 75 who were being treated with antiandrogens and experiencing at least three hot flashes per day. The measurement used was the average weekly score of hot flash symptom severity (HFSSS). The results of this study are not available (45).

In a randomized study, the effect of acupuncture on reducing postoperative pain and lessening painkillers dosage were investigated along with acupuncture effects on the acceleration of the return of intestinal motorics in people undergoing prostatectomy (46). The study included men aged 18 to 70 with prostate cancer and planned open prostatectomy. The intensity of postoperative pain was measured using the Numerical Scale of Evaluation (NRS-11) (46). The results were also measured by the number of the combined amount of painkillers taken in milligrams, the time of the first defecation and the results obtained from the EQ-5D-5L questionnaire. However, the results of the above study have not yet been published (46).

## 4 Discussion

Pain is a complex experience influenced by biological, psychological and social factors (47), three important aspects to take into account in the process of the pain management.

Patients with breast, lung, stomach, colon and prostate cancer experience heightened levels of stress, which can affect the psychological background of pain. Furthermore, there is evidence of the adverse effect of stress in the development of the cancer by releasing stress hormones including cortisol, epinephrine and noradrenaline, which in turn can stimulate the proliferation of cancer cells (48). Reducing the stress level in patients can thus contribute to decreasing the progression of the disease (48).

**Figure 3:**
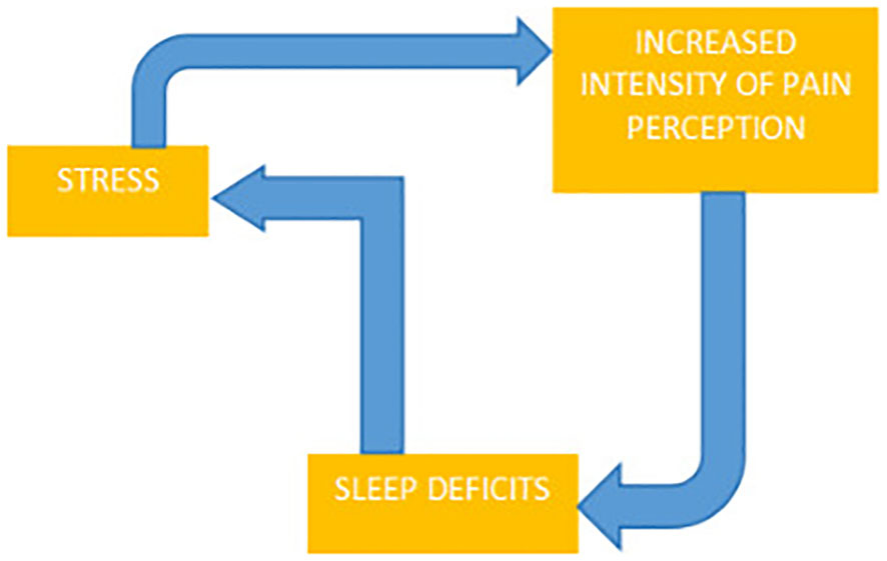
The interrelationships between pain perception, stress, and sleep deficits.

Stress and pain interact on multiple levels, influencing and exacerbating each other, determining relaxation and stress reduction through acupuncture as crucial in chronic pain management. Sleep deficits can lead to increased pain intensity and amplify the stress associated with the disease (**Figure 3**). Acupuncture promotes relaxation, improves sleep quality, and contributes to reducing pain intensity, making it one of the alternative methods for reducing stress in cancer patients and enabling better coping with difficult situations and pain control (15, 49).

Opioids are the preferred medications for alleviating moderate to severe pain in patients with advanced cancer (50). However, one of the most common and significant side effects associated with their use is constipation (51). Additionally, the use of opioids in the treatment of cancer pain can lead to immunosuppressive effects, increasing the risk of infections and weakening the response to treatment. Opioids can also disrupt the functioning of the hormonal system, leading to hypogonadism (52).

It is worth noting that acupuncture can be an effective alternative to opioids in the therapy of cancer pain. Limiting the use of opioids in favor of acupuncture may reduce the risk of experiencing side effects associated with these medications (28). Furthermore, it has been demonstrated that acupuncture can help alleviate the side effects of opioids, such as constipation, thereby reducing the health consequences associated with them (53).

## 5 Conclusions

A review of studies on the use of acupuncture in the treatment of various types of cancer reveals a promising scientific basis for this therapeutic method. Significant conclusions can be drawn from both the analysis of research results and the methodology of the conducted analyses. First and foremost, it is noteworthy that studies on acupuncture in cancer treatment often demonstrate a significant improvement in pain symptoms. It can also reduce the need for opioids and other painkillers, which is beneficial in pain management in cancer patients. For example, studies on gastric, breast, lung, colorectal, and prostate cancers have shown that acupuncture can provide relief from pain, reduce nausea and vomiting, and improve overall quality of life for patients.

The methodology of the conducted studies is also a crucial element of the analysis. It is worth noting that many studies were randomized, placebo-controlled trials, which increases the credibility of the results. Additionally, many studies employed various pain assessment scales and quality of life measures, allowing for a comprehensive evaluation of acupuncture efficacy.

However, there are also methodological limitations, such as the small number of patients in some studies and short observation periods. Furthermore, studies on acupuncture in cancer treatment often consider different stages of the disease, allowing for an assessment of the effectiveness of this method both in the preoperative and postoperative periods. This approach enables a comprehensive evaluation of the impact of acupuncture on the course of the disease and associated symptoms. In the context of further research, it is important to consider the optimal timing and duration of acupuncture therapy, as well as identify patient groups that may benefit most from this form of treatment. Studies should also compare the effectiveness of different acupuncture methods, such as traditional acupuncture needles, electroacupuncture, or transcutaneous electrical acupoint stimulation (TEAS). In summary, studies on the use of acupuncture in cancer treatment show promising results; however, further research is needed to better understand its mechanisms of action and optimal application in clinical practice.
